# Presence of matrix vesicles in the body of odontoblasts and in the inner third of dentinal tissue: A scanning electron microscopyc study

**DOI:** 10.4317/medoral.18650

**Published:** 2013-02-05

**Authors:** Maricela Garcés-Ortíz, Constantino Ledesma-Montes, José Reyes-Gasga

**Affiliations:** 1 Clinical Oral Pathology Laboratory. Facultad de Odontología, Universidad Nacional Autónoma de México. México, 04510, D.F. México; 2 Clinical Oral Pathology Laboratory. Facultad de Odontología, Universidad Nacional Autónoma de México. México, 04510, D.F; 3 New Materials Laboratory. Instituto de Física, Universidad Nacional Autónoma de México. Apartado Postal 20-364. México, 01000, D.F. México

## Abstract

Objectives: The aim of this report is to present the results of a scanning electron microscopic study on the presence of matrix vesicles (MVs) found in human dentine. Study Design: Dentin tissue from 20 human bicuspids was analyzed by means of scanning electron microscopy. Results: MVs were found as outgrowths of the cellular membrane of the odontoblastic body, the more proximal portion of the odontoblastic process before entering the dentinal tubule and in the odontoblastic process within the inner third of the dentin. Size of MVs varied depending on location. In the inner third of dentin, they were seen in diverse positions; as membranal outgrowths, deriving from the odontoblastic process, lying free in the intratubular space and attached to the dentinal wall. Sometimes, they were seen organized forming groups of different sizes and shapes or as multivesicular chains running from the surface of the odontoblastic process to the tubular wall. MVs were present in places never considered: 1) the body of odontoblasts; 2) the most proximal part of the odontoblastic processes before entering the circumpulpal dentine and also: 3) in the inner third of dentinal tissue. Conclusions: According to our results, MVs not only participate during mantle dentin mineralization during early dentinogenesis, they also contribute during the mineralization process of the inner dentin.

** Key words:**Dentin, microvesicles, secretory vesicles, dentin formation, dentin secretion.

## Introduction

Dentin is a mineralized connective tissue with a self recovering capacity and has diverse functions. It surrounds and protects the pulpal tissue, gives support to cementum and provides elastic back up to enamel (1). Dentin tissue is formed by the odontoblasts and their odontoblastic processes (OP) which secrete dentin matrix building the dentinal tubules (DT), the peritubular and the intertubular dentine. Dentin also contains the dentinal fluid some nerves and is classified in primary, secondary and tertiary types. DTs contain the OPs which deposit a complex matrix formed by collagen mainly and non-collagen proteins which later will mineralize (2).

The functional cellular unit of the dentin complex is the odontoblast. This cell is the responsible for the main dentinal functions: dentin formation; including protein secretion and mineral deposition, and with the dentinal fluid, it helps in sensitivity to painful stimuli (2-4). These functions are possible because they contain a complex cellular structure consisting in nucleus, nucleolus and a group of active secretory units; Golghi apparatus, rough endoplasmic reticulum, smooth endoplasmic reticulum and a cytoplasmic prolongation housed within the dentinal tubule named odontoblastic process (1). It is an active cell responsible for secretion and development of dentine, since odontoblast lays down a huge quantity of dentin matrix, builds the main corps of the dental organ and maintains the basic shape of the tooth.

Since the first description by Tomes (5), the cytoplasmic process of the odontoblast has been described as the cytoplasmic extension of the odontoblastic body; it contains cytoskeletal proteins, small mitochondria, specific molecules for endocytosis, lysosomes, is housed within the dentinal tubule and it is separated from the dentinal wall by the periodontoblastic space (6).

Matrix vesicles (MVs) play an important role in mineralization of different mineralizing tissues and they are considered the primary nucleation site. In dentin, MVs are vesicular bodies found within the OP and they were described in the extracellular milieu during the mineralization process of the mantle dentine exclusively (7-13). To date, MVs have not been reported in other dentinal places than mantle of dentine and their morphological features were always studied with transmission electron microscopy.

The aim of this report is to present the results of a scanning electron microscopic (SEM) study on the presence of matrix vesicles found in human dentine.

## Material and Methods

We analyzed 20 caries free human bicuspids. They were obtained from 15 to 21 years old patients undergoing orthodontic treatment. All patients were informed on the objectives of the study and all the 18 years old or older patients and parents of the 17 years old or younger patients signed a Letter of Consent, donating their teeth to our institution for research purposes only. Local anesthesia using Xilocaine with 2% epinephrine were used in all patients and their upper or lower bicuspids were extracted with minimal trauma. The crowns of teeth were separated from roots making a groove at the cemento-enamel junction with water cooled, tungsten carbide bur and a high-speed handpiece. Final separation was made using a chisel and a hammer. Crowns were grooved in mesio-distal direction, split in two halves and immediately immersed in Karnovsky fixative solution at 4°C overnight, rinsed in cacodylate buffer, pH 7.4 and demineralized in 5% nitric acid aqueous solution. After critical point drying, crowns were mounted in aluminum stubs with colloidal silver, coated with a 20 nm-thick gold and examined with a JEOL 2000 SEM (JEOL, Japan).

## Results

**Figure 1:**
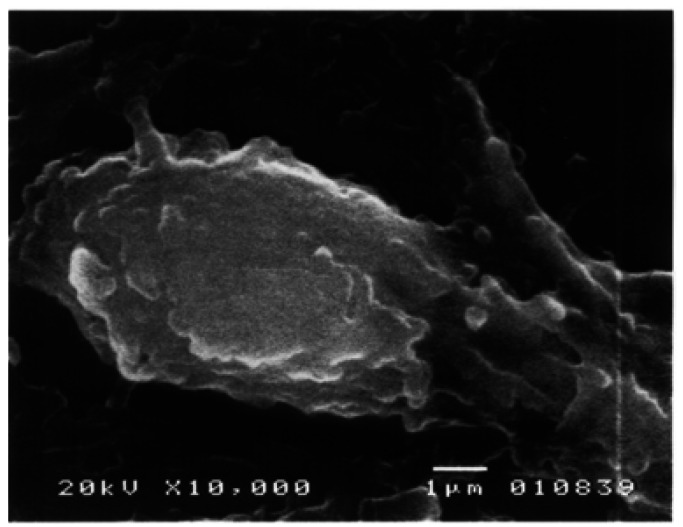
Photomicrograph of an odontoblast with numerous microvesicles bulging from the cell membrane. SEM. 15,000X. Barr. 1µ.

**Figure 2:**
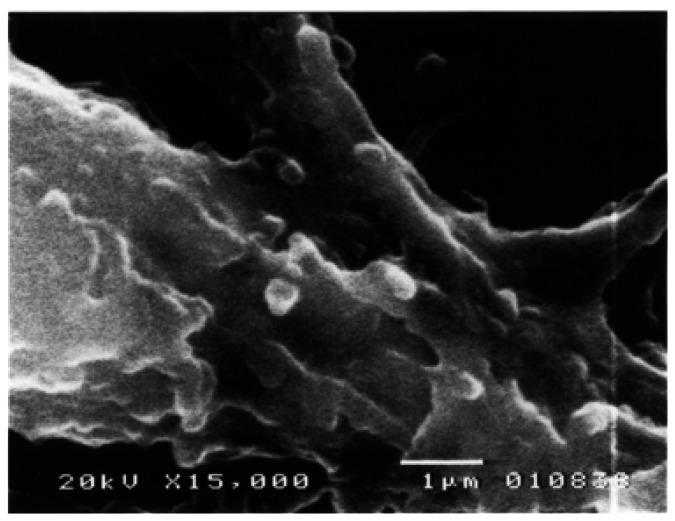
Odontoblastic process (asterisk) just before entering the non-mineralized dentin (star). Numerous vesicles protruding from the cell membrane can be seen (arrows). SEM. 15,000X. Barr. 1µ.

Under SEM, a well developed lining of odontoblasts and a band of non-mineralized pre-dentin were seen. Odontoblasts were observed as elongated cylindrical cells with cytoplasmic prolongations running towards dentin. Some of them presented smooth surfaced cellular bodies, showing numerous round or oval outgrowths of exocytotic appearance protruding the odontoblastic plasmatic cellular membrane, measuring from 0.7 to 1.6µ and covering almost entirely the odontoblastic surface (Fig. 1). These vesicles were also identified in the membrane of the odontoblastic process before entering the dentinal tubule (Fig. 2). In this area, these structures were smaller measuring among 0.28 to 0.72µ.

**Figure 3:**
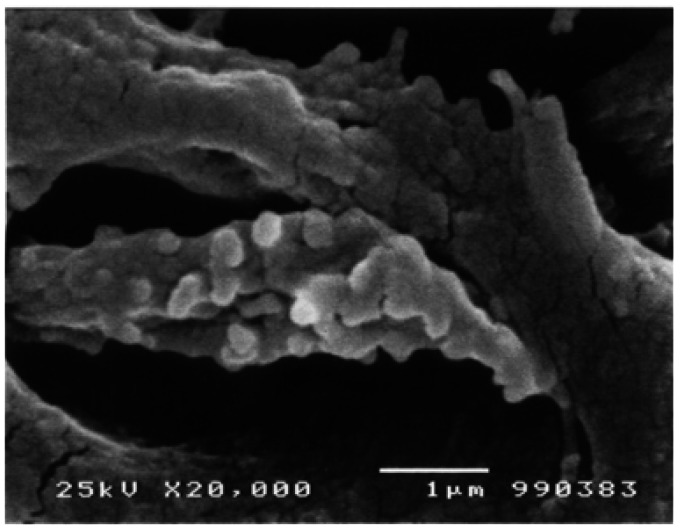
Inner third of dentin. Odontoblastic process with multiple matrix vesicles attached to its surface. Note that peritubular dentine is formed by numerous calcified micronodules. Observe that size of the microvesicles is similar to the dentinal nodules. SEM. 20,000X. Barr. 1µ.

**Figure 4:**
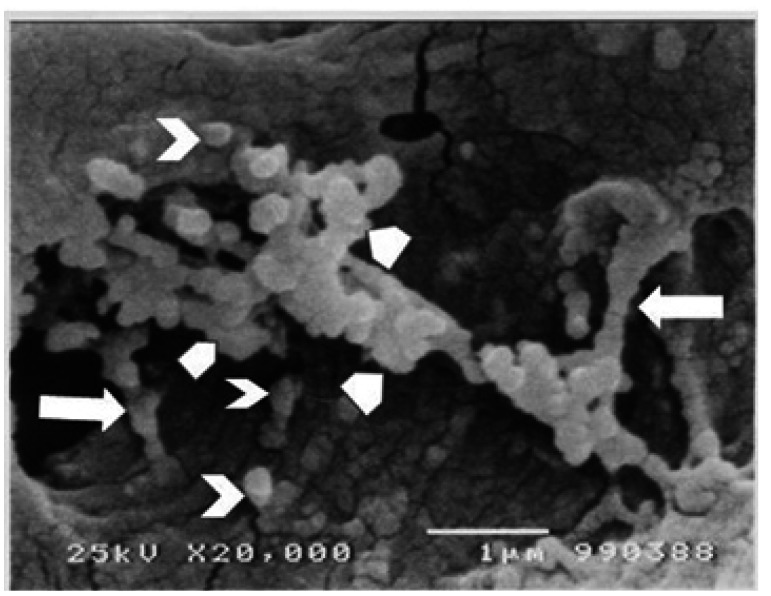
Matrix microvesicles in different arrangements. Some of them forming chains running from the odontoblastic process to the dentin wall (arrows), others are attached to the dentinal wall only (chevron) and some of them are forming multi-vesicular groups (arrow heads). Note that size among vesicles and intra-tubular micronodules is similar. SEM. 20,000X. Barr. 1µ.

Searching through the dentinal tissue, numerous similar structures were found associated to the OPs within DTs at the inner third of the mineralized dentin (Fig. 3). These MVs were vesicular, spherical, smooth surfaced bodies, closely associated to the external portion of the OPs’ cellular membranes measuring from 0.11µ to 0.32µ in diameter. In some instances; their size was very small and they were seen as slight elevations on the surface of the OP or were found as large multivesicular structures, formed by coalescence of several or numerous MVs (Fig. 3). These multivesicular collections were of variable size and shape. In some instances, they formed chains of varying longitude running from the surface of the OP to the tubular wall (Fig. 4). These structures were frequently seen in zones where the peri-tubular dentine showed the presence of rough, granular, spheroidal structures, forming numerous mineralized calcospherites. In places where these vesicles were seen attached the surface of the DT, it was clearly seen that size of the dentinal mineralized calcospherites was similar to that of the vesicles (Fig. 4).

As it is seen in figures 3 and 4, MVs clearly derived from the cytoplasmic membrane of the OP, isolated or in multi-vesicular groups: isolated MVs were seen attached to the surface of the OP; lying “free” in the intratubular periodontoblastic space or attached to the wall of the DT. The MVs forming groups were seen congregated in irregularly shaped multivesicular groups or they were several MVs grouped forming chains with one side attached to the OP and the other side secured to the dentinal wall.

MVs were observed protruding from the cell membrane of the OPs and developing from it by an exocytoticlike mechanism. This finding could suggest they probably represent dentin matrix vesicles associated to the dentinal mineralization process. Careful analysis of the figures 3 and 4, disclosed that DTs containing OPs coupled with the above described vesicular bodies, their walls presented a granular appearance. This finding strongly suggests that these vesicular bodies were matrix vesicles associated to dentinal mineralization. This assumption was confirmed by the fact that the tubular wall was formed by numerous calcosferites. In these areas, these vesicular bodies measured among 0.09 to 0.3µ. Comparatively, tubular calcosferites had a similar size (Figs. 3,4).

## Discussion

MVs were originally discovered during ultrastructural studies in bone and plate cartilage development (14,15) and it is considered they play an important role during mineralization of different mineralizing tissues (16-18). During tooth development, MVs were identified in early dentin mineralization zones only and they are considered the primary nucleation site for the beginning of the mineralization process (7,8,13).

To date, MVs in dentinal tissue were only described in the mantle of dentin during its initial development (7,8,11,12) and never during late dentinal growth. In this paper we present findings showing that MVs can be observed in places not previously considered: 1) the cytoplasmic membrane of mature, active odontoblasts without evident dentin matrix deposition nor association to the mineralization process; 2) MVs were located in the portion of the OP before entering the non-mineralized dentin tissue and 3) they were observed in the inner third of the mature dentin. In the above mentioned locations, the presence of MVs was not previously reported.

Using transmission electron microscopic techniques, MVs appear as vesicular, roundish or spherical bodies with diameter among 50-200nm, they appear covered by a bi-laminar unit membrane and usually contains faintly granular material which sometimes it is needle-like mineralized crystals (8,12). During early tooth development, MVs can be found in the cell bodies of the odontoblastic cells and immersed within the non-mineralized or initially mineralized dentinal matrix (7,8,10-12).

MVs were never seen in dentinal tissue using SEM techniques. In this study we found these vesicular bodies associated to the cytoplasmic membrane of odontoblasts and OPs at different locations showing similar features to MVs described in other mineralizing tissues (16-18). In the surface of the cell membrane of odontoblasts, we found numerous round or oval outgrowths of exocytotic appearance, measuring from 0.7 to 1.6µ and covering almost entirely the cellular surface of the odontoblastic cell body. Similar structures were also observed in the most proximal portion of the OP located outside the recently deposited dentin matrix and compared with those found on the surface of the odontoblasts, they were smaller. Also, MVs were found associated to mantle dentin formation. In this area, these structures were similar in size and shape to those found associated to the odontoblastic bodies and OPs before entering the non-mineralized dentinal matrix. It was a very surprising finding to note that these MVs were also seen associated to OPs in the inner third of the dentinal tissue. These structures clearly derived from the OPs as exocytotic bodies and three growth phases before to reach the mature mineralized dentin were identified: 1) They start as protrusions of different sizes over the OP surface which enlarge and grow by filling with matrix substance, then; 2) they detach from the OP and later; 3) the MVs attach over the wall of the DT and starts the formation of a micronodule. Sometimes, they can be found in the intratubular space, in other instances, they formed chains connecting the OP with the mineralized wall of the DT and at times they were identified forming multivesicular groups. These results strongly suggest that MVs derived from the OP and that may have a function during the deposit of the dentinal matrix in the extracellular space. As it was demonstrated in this study, MVs attached to the inner dentinal wall of the DT had a similar size and shape compared with the mineralized calcosferites composing the corpus of the tubular dentine. This finding suggests that these structures play a role in the mineralization process of the peritubular dentine. Taking together all the findings from this study, we suggest that these vesicular structures are MVs containing secretory material susceptible of mineralization in the form of calcosferites and that they are not only involved in the early dentin formation during development of the mantle of dentin. These results also suggest that these MVs play an active role in the late dentinal development and mineralization processes.

It is well known that dentin is formed by direct deposit of the proteic and non-proteic components of the dentinal matrix to the extra-cellular space with the concomitant mineralization (7,10,) and that this phenomenon does not involve the presence of MVs. Our results suggest that another mineralization process occurs during dentin development. It includes MV formation, detachment from the OP, attachment to the DT wall and mineralization of the dentin matrix within the MV. This contention is supported by the fact that MVs attach to the dentinal wall and their similarity in size and shape with the neighbor mineralized calcospherites located in the peritubular dentine. This is contention is also supported by the findings of Agematsu et al (9), they found numerous mineralized bodies surrounded by a membrane, containing polyhedral crystals attached to dentinal canals in human deciduous dentin.

We like to point out that it is necessary to add new knowledge related to the findings presented in this report.
